# ABA-mediated regulation of leaf and root hydraulic conductance in tomato grown at elevated CO_2_ is associated with altered gene expression of aquaporins

**DOI:** 10.1038/s41438-019-0187-6

**Published:** 2019-09-11

**Authors:** Liang Fang, Lamis Osama Anwar Abdelhakim, Josefine Nymark Hegelund, Shenglan Li, Jie Liu, Xiaoying Peng, Xiangnan Li, Zhenhua Wei, Fulai Liu

**Affiliations:** 1 Department of Plant and Environmental Sciences, Faculty of Science, University of Copenhagen, Højbakkegaard Alle 13, 2630 Taastrup, Denmark; 2 Key Laboratory of Agricultural Soil and Water Engineering in Arid and Semiarid Areas, Ministry of Education, Northwest A&F University, 712100 Yangling, Shaanxi China; 3 College of Bioscience and Biotechnology, Hunan Agricultural University, 410128 Changsha, Hunan China; 4 Key Laboratory of Mollisols Agroecology, Northeast Institute of Geography and Agroecology, Chinese Academy of Sciences, 130102 Changchun, China; 5 Present Address: Centre for Crop Systems Analysis, Department of Plant Sciences, Wageningen University & Research, PO Box 430, 6700 AK Wageningen, The Netherlands

**Keywords:** Stomata, Plant physiology

## Abstract

Elevated CO_2_ concentration in the air (*e*[CO_2_]) decreases stomatal density (SD) and stomatal conductance (*g*_s_) where abscisic acid (ABA) may play a role, yet the underlying mechanism remains largely elusive. We investigated the effects of *e*[CO_2_] (800 ppm) on leaf gas exchange and water relations of two tomato (*Solanum lycopersicum*) genotypes, Ailsa Craig (WT) and its ABA-deficient mutant (*flacca*). Compared to plants grown at ambient CO_2_ (400 ppm), *e*[CO_2_] stimulated photosynthetic rate in both genotypes, while depressed the *g*_s_ only in WT. SD showed a similar response to *e*[CO_2_] as *g*_s_, although the change was not significant. *e*[CO_2_] increased leaf and xylem ABA concentrations and xylem sap pH, where the increases were larger in WT than in *flacca*. Although leaf water potential was unaffected by CO_2_ growth environment, *e*[CO_2_] lowered osmotic potential, hence tended to increase turgor pressure particularly for WT. *e*[CO_2_] reduced hydraulic conductance of leaf and root in WT but not in *flacca*, which was associated with downregulation of gene expression of aquaporins. It is concluded that ABA-mediated regulation of *g*_s_, SD, and gene expression of aquaporins coordinates the whole-plant hydraulics of tomato grown at different CO_2_ environments.

## Introduction

Stomata controls the photosynthesis (*A*_n_) and transpiration rates. The ability of plants to regulate the stomatal conductance (*g*_s_), through either modulating the aperture of the stomatal pore in a short term or changing the stomatal density (SD) in a long term, is crucial for their survival in an ever-changing environment. Among other environmental factors, the rising CO_2_ concentration ([CO_2_]) in the atmosphere will have profound impacts on plant physiological processes, particularly those related to stomatal control of leaf gas exchange and plant water relations^1^.

The influences of CO_2_ elevation (*e*[CO_2_]) on stomatal morphology and physiology have been well documented^1–5^. Accumulated evidence showed that *e*[CO_2_] reduces SD^6–9^. It has been suggested that reduction in SD caused by *e*[CO_2_] could be modulated by abscisic acid (ABA) levels^10,11^. Earlier studies have shown that SD correlates positively with plant ABA level^12–14^. However, whether such a relationship also exists for plants grown in different CO_2_ environments remains unknown. The low SD of plants grown at *e*[CO_2_] could curtail the maximal *g*_s_ in a long term, while an immediate reduction of *g*_s_ after exposure to *e*[CO_2_] has often been observed^4,15^. Guard cells could sense the change of [CO_2_] growth environment through responding to intercellular [CO_2_] (C_i_) and not leaf surface [CO_2_]^16^. *e*[CO_2_] has been found to affect several ion channel activities, which may cause depolarization of the guard cell membrane potential^4^. In addition, ABA could play an important role in inducing stomatal closure in plants grown under *e*[CO_2_]^10,17^. An earlier study showed that ABA could enhance the response of stomata to changes of [CO_2_]^18^. More recently, literature revealed that *e*[CO_2_]-caused closure of stomata might be mediated by ABA^11^. On the other hand, a recent study reported that *e*[CO_2_]-induced stomatal closure is ABA independent via modulating OST1/SnRK2 kinases^19^. Therefore, the role of ABA in mediating *g*_s_ response to *e*[CO_2_] merits further investigations. Moreover, it is well recognized that the distribution of ABA in plants is affected by the apoplast pH^20^, which could be affected by the CO_2_ growth environment hence modulating the efficiency of the ABA-mediated stomatal response to *e*[CO_2_]. However, until now this aspect has not been explored.

Many researchers have reported that plants grown at *e*[CO_2_] could maintain higher (less negative) leaf water potential (*Ψ*_l_), which could be partially attributed to the lowered *g*_s_ and hence transpiration rate at *e*[CO_2_]^5,21^. Nevertheless, higher *Ψ*_l_ of plants grown at *e*[CO_2_] was not always the case, even though *g*_s_ and transpiration rate were found to be lower, but hydraulic conductance could also be reduced in plants grown at *e*[CO_2_]^22,23^, which may offset the positive effect of lowered *g*_s_ and transpiration rate on *Ψ*_l_. Moreover, the response of plant hydraulic conductance to *e*[CO_2_] was variable as controversial results were reported^21^. The changes of hydraulic conductance may be associated with changes of the abundance or activity of aquaporins that control plasma membrane water permeability^24–26^. Yet, it remains largely unknown whether *e*[CO_2_] affects the expression of genes encoding aquaporins in leaf and root and whether endogenous ABA is involved in this process.

This study aimed to investigate the responses of leaf gas exchange, water relation characteristics, and hydraulic conductance of tomato plants to *e*[CO_2_]. To achieve this, two tomato genotypes (GEs) differing in the endogenous ABA level were tested. We hypothesized that ABA would exert an important role in mediating the responses of stomatal behavior and plant water status to *e*[CO_2_] by modulating both stomatal aperture and SD as well as the expression of aquaporins and thereby the whole-plant hydraulics and water balance.

## Results

The ABA-deficient *flacca* tomato had significantly small leaf area and shoot biomass in relation to the wild-type (WT) plants; although *e*[CO_2_] tended to increase the growth for both of the GEs, the increments were not statistically significant (Fig. S1).

### Leaf gas exchange

**Fig. 1:**
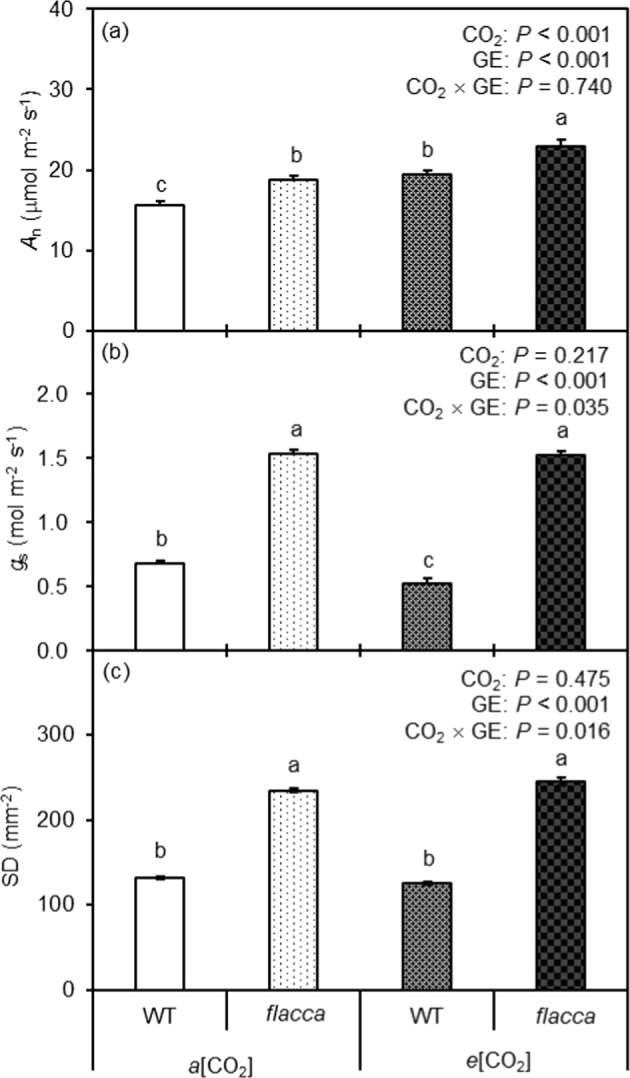
Leaf gas exchange and stomatal morphology response of the two tomato genotyes to different CO_2_ growth environments. Net photosynthetic rate (*A*_n_) (**a**), stomatal conductance (*g*_s_) (**b**), and stomatal density (SD) (**c**) of wild-type tomato “Ailsa Craig” (WT) and its respective ABA-deficient mutant (*flacca*) grown at ambient (400 ppm, *a*[CO_2_]) and elevated (800 ppm, *e*[CO_2_]) atmospheric CO_2_ concentrations. The effects of CO_2_ growth environment (CO_2_) and genotype (GE) as well as their interactions CO_2_ × GE are presented (two-way ANOVA). The different letters on the columns indicate statistically significant difference between the treatments by Tukey’s test at *P* < 0.05. Error bars indicate standard error of the means (SE) (*n* = 8)

Compared to WT, *flacca* had significantly higher *A*_n_ and *g*_s_ under both CO_2_ growth conditions (Fig. 1a, b). The *A*_n_ of both WT and *flacca* were significantly higher in the *e*[CO_2_] plants than in the *a*[CO_2_] plants. In relation to plants grown at *a*[CO_2_], a reduction of *g*_s_ at *e*[CO_2_] was only noticed in WT and not in *flacca*.

### Stomatal density

Significantly higher SD in *flacca* than in WT was noticed across the two CO_2_ growth environments. Compared to the *a*[CO_2_] plants, SD tended to be lower when grown at *e*[CO_2_] for WT (although not statistically significant), whereas for *flacca* a slight increase of SD was noticed in plants grown at *e*[CO_2_], resulting in a significant interaction between CO_2_ and GE (Fig. 1c).

### Leaf and xylem sap ABA concentration

**Fig. 2:**
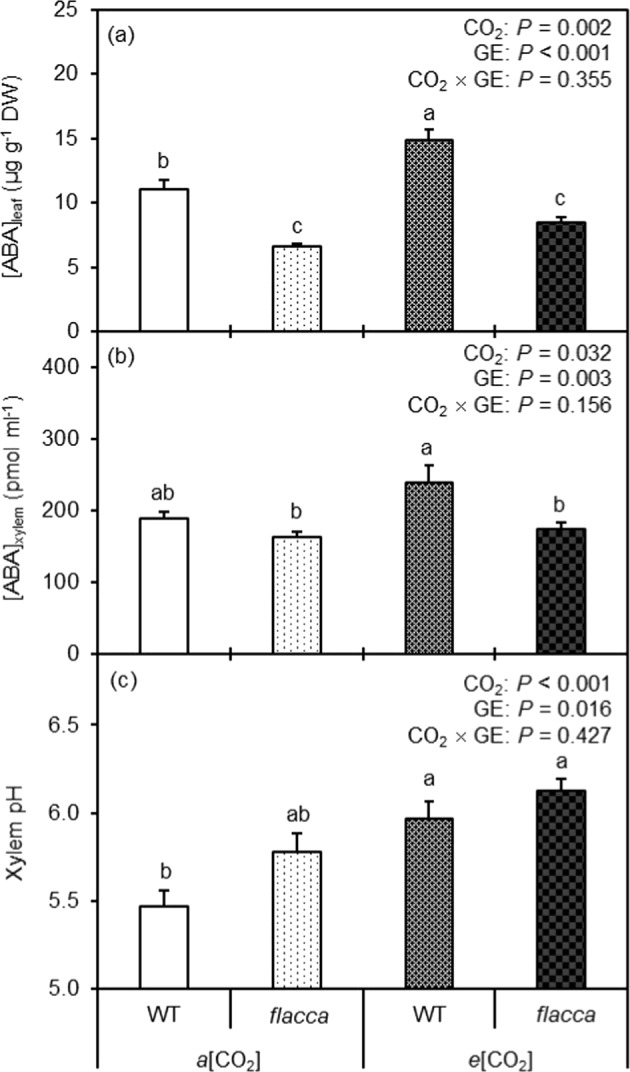
Leaf and xylem sap ABA concentration and xylem sap pH of the two genotypes of tomato as affected by different CO_2_ growth environments. Leaf ABA concentration ([ABA]_leaf_) (**a**), xylem sap ABA concentration ([ABA]_xylem_) (**b**), and xylem pH (**c**) of wild-type tomato “Ailsa Craig” (WT) and its respective ABA-deficient mutant (*flacca*) grown under ambient (400 ppm, *a*[CO_2_]) and elevated (800 ppm, *e*[CO_2_]) CO_2_ environments. The effects of CO_2_ growth environment (CO_2_) and genotype (GE) as well as their interactions CO_2_ × GE are presented (two-way ANOVA). The different letters on the columns indicate statistically significant difference between the treatments by Tukey’s test at *P* < 0.05. Error bars indicate standard error of the means (SE) (*n* = 8)

As expected, significantly higher leaf and xylem ABA concentrations were observed in WT compared to *flacca* (Fig. 2a, b). In relation to the *a*[CO_2_] plants, *e*[CO_2_] significantly increased [ABA]_leaf_ and [ABA]_xylem_, while the magnitude of increase was greater in WT than in *flacca*, although no significant CO_2_ × GE effect was found.

### Xylem sap pH

The *e*[CO_2_] plants had higher xylem pH than the *a*[CO_2_] plants; and in general *flacca* had higher xylem pH than WT irrespective to the CO_2_ growth environments (Fig. 2c).

**Fig. 3:**
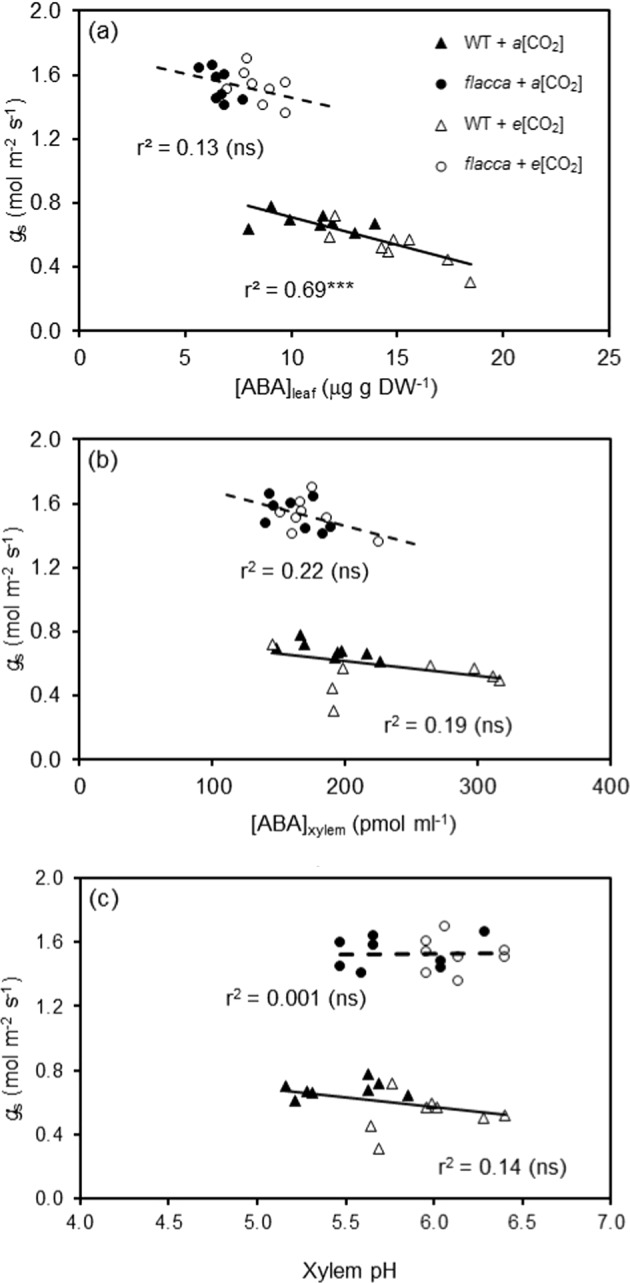
Correlations between stomatal conductance to leaf and xylem ABA concentration and xylem sap pH of the two genotypes of tomato grown at different CO_2_ levels. Correlations of stomatal conductance (*g*_s_) to leaf ABA concentration ([ABA]_leaf_) (**a**) and xylem sap ABA concentration ([ABA]_xylem_) (**b**), and xylem pH (**c**) of wild-type tomato “Ailsa Craig” (WT) and its respective ABA-deficient mutant (*flacca*) grown under ambient (400 ppm, *a*[CO_2_]) and elevated (800 ppm, *e*[CO_2_]) CO_2_ environments. Triple asterisks (***) indicates that the regression line is statistically significant (*P* < 0.001) and ns denotes no significance

For WT, *g*_s_ was negatively correlated with [ABA]_leaf_ across the two CO_2_ growth environments (*P* < 0.001); although a similar relationship was also noticed in *flacca*, the linear regression was not statistically significant (Fig. 3a). Likewise, negative linear relationships between [ABA]_xylem_ and *g*_s_ was observed across the two CO_2_ growth environments for both GEs; the linear regressions, however, were not statistically significant (Fig. 3b). No obvious relationship between xylem pH and *g*_s_ were evident (Fig. 3c).

### Plant water relations

**Fig. 4:**
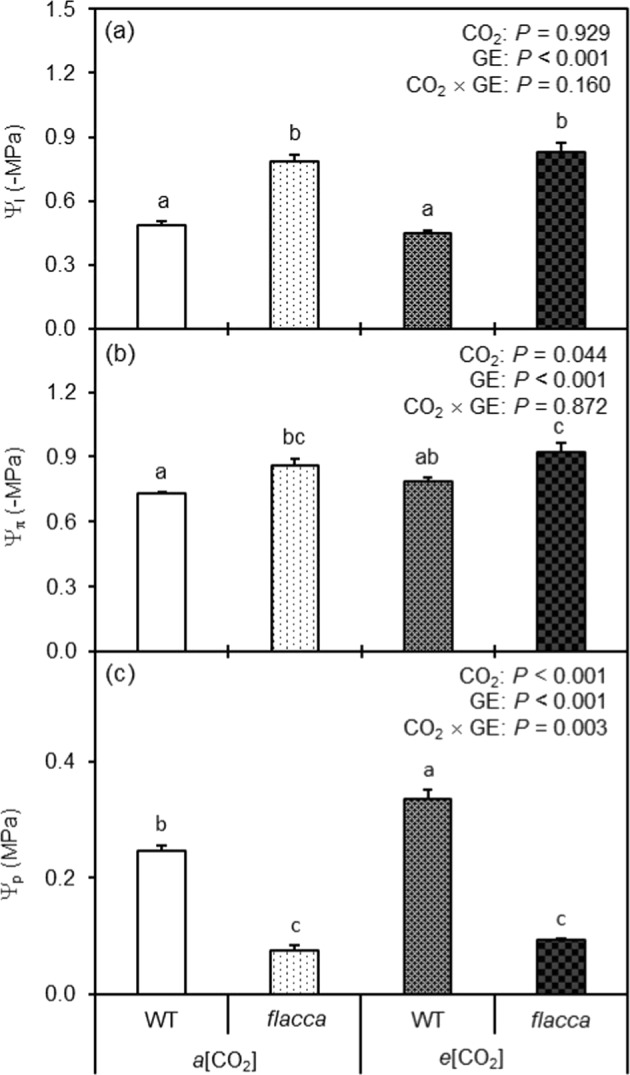
Leaf water relation characteristics of the two tomato genotypes as affected by different CO_2_ growth environments. Leaf water potential (*ψ*_l_) (**a**), osmotic potential (*Ψ*_π_) (**b**), and turgor pressure (*Ψ*_p_) (**c**) of wild-type tomato “Ailsa Craig” (WT) and its ABA-deficient mutant (*flacca*) grown under ambient (400 ppm, *a*[CO_2_]) and elevated (800 ppm, *e*[CO_2_]) CO_2_ environments. The effects of CO_2_ growth environment (CO_2_) and genotype (GE) as well as their interactions CO_2_ × GE are presented (two-way ANOVA). The different letters on the columns indicate significant difference between the treatments by Tukey’s test at *P* < 0.05. Error bars indicate standard error of the means (SE) (*n* = 8)

*flacca* had lower (more negative) *Ψ*_l_ and *Ψ*_π_ and lower *Ψ*_p_ compared to WT (Fig. 4). CO_2_ growth environment had no effect on *Ψ*_l_, while *e*[CO_2_] decreased *Ψ*_π_ as compared to *a*[CO_2_] (Fig. 4b). *e*[CO_2_] increased the *Ψ*_p_ of WT but not of *flacca* (Fig. 4c).

### Hydraulic conductance

**Fig. 5:**
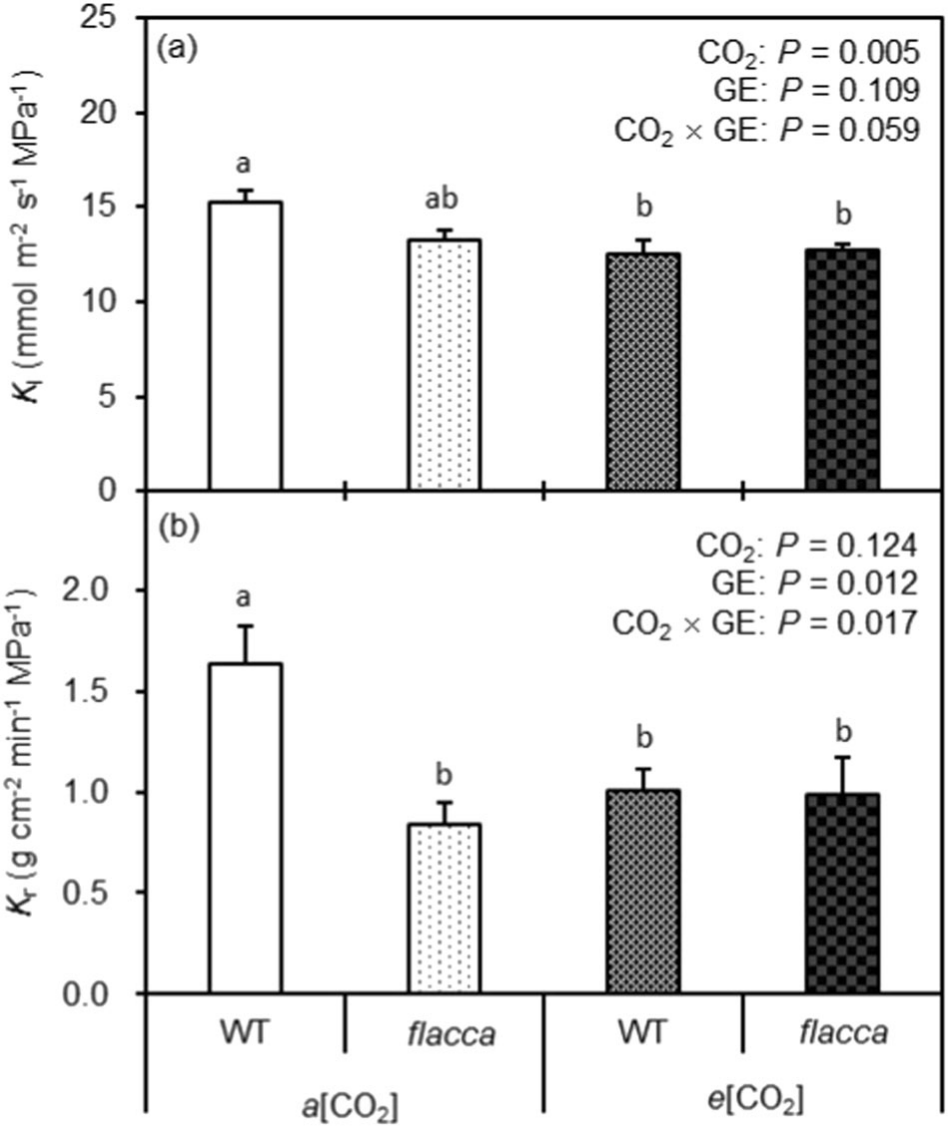
Leaf and root hydraulic conductance of the two tomato genotypes as affected by different CO growth environments. Leaf hydraulic conductance (*K*_l_) (**a**) and root hydraulic conductance (*K*_r_) (**b**) of wild-type tomato “Ailsa Craig” (WT) and its representative ABA-deficient mutant (*flacca*) grown under ambient (400 ppm, *a*[CO_2_]) and elevated (800 ppm, *e*[CO_2_]) CO_2_ environments. The effects of CO_2_ growth environment (CO_2_) and genotype (GE) as well as their interactions CO_2_ × GE are presented (two-way ANOVA). The different letters on the columns indicate significant difference between the treatments by Tukey’s test at *P* < 0.05. Error bars indicate standard error of the means (SE) (*n* = 4)

Compared to the *a*[CO_2_] plants, lower *K*_l_ when grown at *e*[CO_2_] was observed (Fig. 5a); however, the reduction was less significant in *flacca* than in WT resulting in a significant interaction between CO_2_ and GE. The *K*_r_ of WT was significantly higher than that of *flacca* when grown at *a*[CO_2_], whereas they had a similar *K*_r_ when grown at *e*[CO_2_] (Fig. 4b). *e*[CO_2_] decreased *K*_r_ only in WT while it slightly increased *K*_r_ in *flacca* in relation to the plants grown at *a*[CO_2_] (Fig. 5b)

### Expression of genes encoding aquaporins of the plasma membrane intrinsic protein (PIP) subgroup

**Fig. 6:**
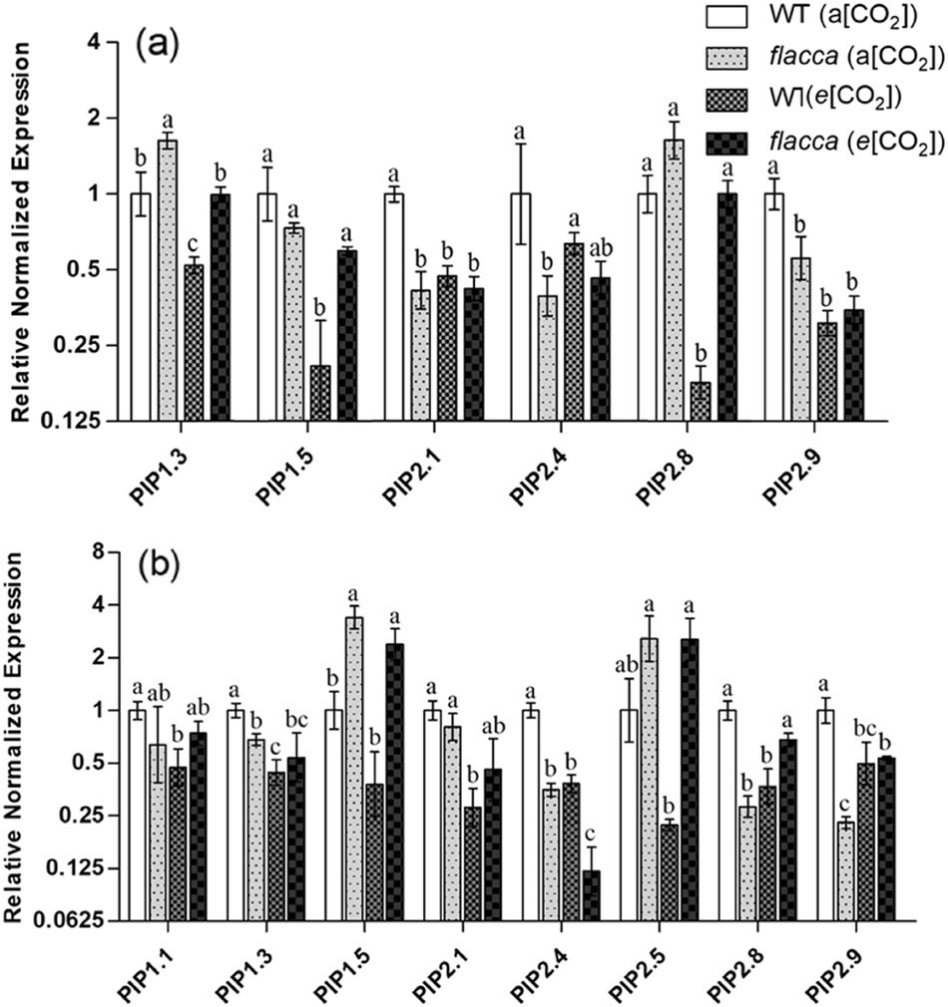
Effect of different CO_2_ growth environments on the relative gene expression of aquaporins in leaf and root of the two tomato genotypes. Relative expression of genes encoding the aquaporin subfamily of plasma membrane intrinsic proteins (PIPs) in leaf (**a**) and root (**b**) of wild-type tomato “Ailsa Craig” (WT) and its representative ABA-deficient mutant (*flacca*) grown under ambient (400 ppm, *a*[CO_2_]) and elevated (800 ppm, *e*[CO_2_]) CO_2_ environments. Different letters on the top of the columns for each PIP gene indicate significant difference between the treatments by Tukey’s test at *P* < 0.05. Error bars indicate standard error of the means (SE) (*n* = 4)

In leaves of WT, transcripts of four PIPs (*PIP1.5*, *PIP2.1*, *PIP2.8*, and *PIP2.9*) responded to *e*[CO_2_] with a 2–5-fold downregulation of expression levels (Fig. 6a). *PIP1.3* and *PIP2.4* showed similar trends but were not significant or below the twofold change cut-off. In *flacca*, PIPs showed only minor fluctuations in transcript levels none of which were significant when comparing *a*[CO_2_] to *e*[CO_2_] growth conditions. When comparing leaf PIP expression between the two GEs grown at *a*[CO_2_], WT showed significantly higher *PIP2.1*, *PIP2.4* and *PIP2.9* expression than *flacca*.

In roots of WT, PIP transcript responses to *e*[CO_2_] showed a similar response as in leaves. All 8 root PIPs showed 2–4-fold downregulation in response to *e*[CO_2_]; however, just transcriptional changes in 5 PIPs were found to be significant (*PIP1.3*, *PIP2.1*, *PIP2.4*, *PIP2.5*, and *PIP2.8*) (Fig. 6b). As in leaves, PIPs of *flacca* did not follow the clear response observed in WT. Five *flacca* root PIPs did not respond in transcript abundance to the *e*[CO_2_] growth environment. However, three PIP transcripts responded with significant twofold upregulation (*PIP2.8* and *PIP2.9*) or downregulation (*PIP2.4*) in *flacca* grown at *e*[CO_2_] in relation to that grown at *a*[CO_2_]. Furthermore, at *a*[CO_2_] *PIP1.3*, *PIP2.4*, *PIP2.8*, and *PIP2.9* had significantly lower expression level while *PIP1.5* had significantly higher expression level, respectively, in *flacca* than in WT (Fig. 6b).

## Discussion

It is well known that *e*[CO_2_] enhances *A*_n_ while reducing *g*_s_, although the response may vary among species and different growth environments^4,27^. Consistent with this, here *A*_n_ was stimulated by *e*[CO_2_] in both WT and *flacca* plants (Fig. 1a); however, reduction of *g*_s_ by *e*[CO_2_] was only observed in the WT and not in *flacca* (Fig. 1b). As expected, at both CO_2_ growth environments, WT plants possessed significantly greater [ABA]_leaf_ and [ABA]_xylem_ than *flacca* (Fig. 2a, b); also, *e*[CO_2_] increased [ABA]_leaf_ and [ABA]_xylem_ more pronounced in WT than in *flacca*. For WT, *g*_s_ was negatively correlated with [ABA]_leaf_ (Fig. 3a), revealing that *g*_s_ was most probably controlled by [ABA]_leaf_ across the two CO_2_ growth environments. Such relationship, however, was not evident for [ABA]_xylem_ and *g*_s_, although earlier studies have frequently reported that *g*_s_ correlated better with [ABA]_xylem_ than with [ABA]_leaf_^28^. Besides, for the two GEs the change of *g*_s_ in response to *e*[CO_2_] was associated with a similar pattern of change in SD (Fig. 1c), suggesting that the endogenous ABA level exerted an important role in the *e*[CO_2_]-induced modulation of SD and thus *g*_s_. Therefore, it is reasonable to postulate that the endogenous ABA level had influenced the responsiveness of SD and *g*_s_ to *e*[CO_2_] in tomato plants. In line with this, it has been reported that *e*[CO_2_]-induced stomatal closure and reductions in SD was modulated by plant ABA levels^10,11^. However, a positive correlation between SD and plant ABA level previously reported in other studies is contradictory to results obtained here^12–14^. Moreover, in addition to ABA, cytokinins and other phytohormones could have also been involved in stomatal regulation in plants grown at *e*[CO_2_]^29^.

An earlier study suggested that the higher [ABA]_leaf_ in the *e*[CO_2_] plants might be caused by slight osmotic stress due to the relative higher solutes’ accumulation induced by rising *A*_n_ when plants grow at *e*[CO_2_]^30^. This was seemingly true here as *e*[CO_2_] led to more negative *Ψ*_π_ in all plants (Fig. 4b). However, ABA synthesis in leaf is believed to be linked with *Ψ*_p_^31^, and an increased *Ψ*_p_ in plants grown at *e*[CO_2_] (Fig. 3c) would result in a low [ABA]_leaf_, disagreeing with the results of the present study. Recently, evidence has indicated that ABA accumulation in drying leaves is due to a decrease in cell volume, not due to reduction of *Ψ*_p_^32^. Moreover, the greater [ABA]_xylem_ of the *e*[CO_2_] plants could be linked to their higher xylem sap pH in relation to the *a*[CO_2_] plants (Fig. 2b, c)^33^. Besides, the lowered root hydraulic conductance at *e*[CO_2_] could also contribute to the greater [ABA]_xylem_ in the *e*[CO_2_] plants^22^, assumingly attributed to a reduced rate of sap flow during collection, which may cause a concentration effect on the xylem sap. Our results disagree with Li et al., who reported that *e*[CO_2_] did not affect [ABA]_leaf_ in tomato plants^34^, and the reasons behind this disagreement are unknown, which merit further studies.

In literature, very little information is available about how *e*[CO_2_] influences xylem sap pH. In this study, higher xylem sap pH was observed in the *e*[CO_2_] plants compared to the *a*[CO_2_] plants (Fig. 2c), suggesting that xylem sap pH was affected by [CO_2_]. This is a novel finding, although the mechanisms behind remain speculative. One mechanism could be due to the bicarbonate ion (HCO_3_^−^), which is produced when CO_2_ dissolve in xylem sap that modulates the pH. Another mechanism might be linked to a disturbed root ion (e.g., nitrate) uptake caused by *e*[CO_2_]^35^; a reduced nitrate uptake under *e*[CO_2_] would result in an increase of xylem pH as suggested by a previous study^36^. Interestingly, compared to WT plants, *flacca* had greater xylem sap pH (Fig. 2c); this contradicts the common consensus that a high xylem sap pH would enable more efficient stomatal closure^33^, yet the reasons behind this are unknown. As mentioned previously, an increased xylem sap pH could retain ABA in the apoplast thereby more efficiently inducing stomatal closure^20,33,37^. Here, in addition to the contribution of a slightly lowered SD, the *e*[CO_2_]-induced reduction in *g*_s_ in the WT could be partially ascribed to the higher [ABA]_leaf_ and/or [ABA]_xylem_ as well as a greater xylem sap pH.

Accumulated evidence indicates that changes in *g*_s_ could lead to changes in *Ψ*_l_ by altering the transpiration rate in plants under well-watered conditions^38^. In the present study, the greater *g*_s_ of *flacca* could have resulted in lower *Ψ*_l_, and vice versa for the WT plants (Fig. 3a), consistent with previous findings in the same GE^39^. Early studies have indicated that *e*[CO_2_] could lead to a higher *Ψ*_l_ in plants^5,23^. In agreement with this, the *Ψ*_l_ of WT plants was slightly higher (less negative) under *e*[CO_2_] than at *a*[CO_2_], though the overall *e*[CO_2_] effect on *Ψ*_l_ was not statistically significant (Fig. 4a). In addition, *e*[CO_2_] decreased *Ψ*_π_ in all plants affirming our earlier findings in tomato^5^; while *flacca* had significantly lower *Ψ*_π_ than WT under both CO_2_ growth environments (Fig. 3b), which could be a result of enhanced solutes’ accumulation caused by the greater photosynthetic rate (*A*_n_) in those plants (Fig. 1a). Also, a higher [ABA]_leaf_ might induce greater vacuolar invertase activity in the leaf, which could enhance hexose concentrations thereby contributing to a lowered *Ψ*_π_^40^. The significantly greater *Ψ*_p_ in the *e*[CO_2_] plants was most likely a consequence of the lowered *Ψ*_π_ as the *Ψ*_l_ was almost unaffected by CO_2_ growth conditions. Further, it was noticed that the *Ψ*_p_ of *flacca* was much lower than that of WT tomato at both CO_2_ growth conditions (Fig. 4c) and that could be attributed to the relatively greater dehydration of the leaf caused by the greater *g*_s_ in *flacca*.

Several early studies have demonstrated that plant hydraulic conductance was reduced when grown at *e*[CO_2_]^22,41^. In line with this, here the *e*[CO_2_] plants possessed significantly lower *K*_l_ and *K*_r_ in WT (Fig. 4a, b). The change of hydraulic conductance of WT plants grown at *e*[CO_2_] was closely associated with the change of *g*_s_, indicating that the reduction in hydraulic conductance could be due to a homeostatic adjustment by the plants in order to match hydraulic conductance with the lowered *g*_s_ at *e*[CO_2_]^42^. However, this was not the case in *flacca*, where the *K*_l_ and *K*_r_ were almost identical at both CO_2_ growth environments (even a slight increase of *K*_r_ of the *e*[CO_2_] plants as compared to the *a*[CO_2_] plants) (Fig. 5a, b). Moreover, in the present study, *flacca* had lower *K*_l_ and *K*_r_ compared to WT under *a*[CO_2_] (Fig. 4a, b). This was in agreement with earlier findings that a higher endogenous ABA level linked to a greater hydraulic conductance^43–45^. Recently, a study also reported that in barley the ABA-deficit mutant possessed significantly lower hydraulic conductance as compared with the WT^46^. These authors suggested that high ABA level and hence greater aquaporin abundance and higher hydraulic conductivity seem essential to sustain the *Ψ*_l_ in barley plants. Nonetheless, although the endogenous ABA level was greater in WT plants grown at *e*[CO_2_] than at *a*[CO_2_] (Fig. 2a, b), the hydraulic conductance was lower in those plants (Fig. 5a, b), indicating that, beside endogenous ABA, other factors might also be involved in the modulation of plant hydraulic conductance under *e*[CO_2_].

To explore the mechanisms underlying the *e*[CO_2_]-induced changes in leaf and root hydraulic conductance, the expression of gene encoding major PIP aquaporins were investigated. To date, there is no information available about how *e*[CO_2_] affects the gene expression of aquaporins in tomato plants. A study^26^ suggested that the changes in aquaporins expression could be regulated by CO_2_, which might contribute to the changes of hydraulic conductance in soybean plants, but there was no direct evidence given in the paper. Here in WT plants, genes encoding five out of six and eight PIPs in leaf and root, respectively, were constantly and significantly downregulated by growing at *e*[CO_2_] (Fig. 6). Consistent with this, a study in broccoli (*Brassica oleracea* L. var Italica) showed that *e*[CO_2_] decreased the abundance of PIP1 and PIP2 protein in both leaf and root as compared to *a*[CO_2_]^47^. Similarly, in tobacco (*Nicotiana tabacum*) leaves a downregulation of *NtPIP2;1* gene expression was noticed when grown at *e*[CO_2_]^48^. However, this was not the case for *flacca* where most of the genes were unaffected or even upregulated by *e*[CO_2_], revealing that the endogenous ABA level exerts a crucial role in mediating the response of aquaporins to *e*[CO_2_]. In line with this, several earlier studies demonstrated that ABA is involved in modulating gene expression of PIPs^44,49^. For instance, a study showed that PIPs were upregulated in response to elevated ABA level in *Arabidopis thaliana*^49^; likewise, another study reported that PIPs were downregulated in response to low endogenous ABA level in transgenic maize plants with silenced ABA synthesis^44^. Most interestingly, the changes of aquaporin gene expression coincided well with the changes in *K*_l_ and *K*_r_, indicating that modulation of the gene expression of aquaporins in the leaf and root contributed essentially to the changes of hydraulic conductance in the *e*[CO_2_] plants. The mechanisms underlying such root and shoot coordination in controlling water balance via modulating PIP expression of plants grown at *e*[CO_2_] remain unknown; the modified N nutrition could be involved as suggested by a recent study^50^. Nonetheless, this finding is of great significance for improving our understanding about the responses of tomato plants to *e*[CO_2_] and the role of ABA in mediating these responses.

Taken together, the results of this study reveal that endogenous ABA is involved in modulating the physiological responses of tomato plants to *e*[CO_2_]. ABA-mediated regulation of *g*_s_ and *K*_l_ and *K*_r_ coordinates the whole-plant hydraulics and water balance of tomato plants under different CO_2_ growth environments.

## Materials and methods

### Plant material and growth conditions

Seeds of isogenic WT (cv. Ailsa Craig) tomato and an ABA-deficient tomato mutant (*flacca*) (*Solanum lycopersicum*) were provided by the Lancaster Environment Centre (Lancaster University, UK). The *flacca* is impaired in the oxidation of ABA-aldehyde to ABA thus possessing significantly lower (ca. 20-folds less) endogenous ABA concentrations than WT^51,52^. All potted plants were grown in a climate-controlled greenhouse at Taastrup campus of University of Copenhagen, Denmark (55°67′ N, 12°30′ Ε). The seeds were sown in 4 L pots filled with 2,600 g of peat material (Plugg-och Såjord-Dry matter ca.110 kg m^−3^, organic matter >95%, pH 5.5–6.5 and EC 1.5–2.5 mS cm^−1^) on February 7, 2018. In total, 32 pots were established. Four weeks after sowing, fertilizers were added together with irrigation water in the form of NH_4_NO_3_ (2.8 g) and H_2_KPO_4_ (3.5 g) per pot to avoid any nutrient deficiency.

After sowing, the plants were grown in two separated greenhouse cells (cell 1 and cell 2) with different atmospheric CO_2_ concentrations: ambient (400 ppm, *a*[CO_2_]) and elevated (800 ppm, *e*[CO_2_]), respectively. In each cell, 16 plants (8 WT and 8 *flacca*) were randomly distributed on a growth table. The CO_2_ was enriched inside the cell by emission of pure CO_2_ at one point from a bottle tank and distributed through the ventilation system. The [CO_2_] was monitored every 6 s by a CO_2_ Transmitter (Series GMT220, Vaisala Group, Helsinki, Finland).

**Fig. 7:**
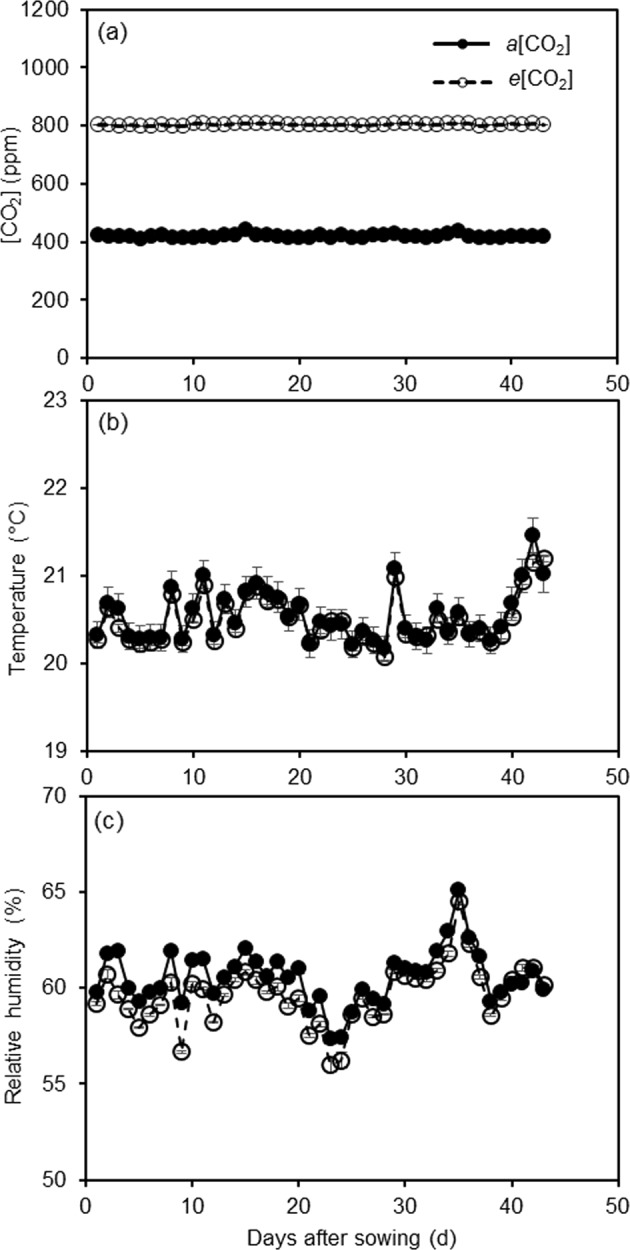
The daily average of atmospheric CO_2_ concentration [CO_2_], air temperature, and relative humidity in the two greenhouse cells during the experimental period. Error bars indicate standard error of the mean (SE = 96)

The day/night air temperature in the both greenhouse cells were set at 20/18 ± 2 °C, relative humidity at 60 ± 2%, photoperiod at 16 h, and photosynthetic active radiation (PAR) at >250 μmol m^−2^ s^−1^ supplied by sunlight plus LDE lamps. The vapor pressure deficit ranged from 0.8 to 1 kPa. The climate data were monitored every 5 min and recorded by a climate computer. The daily average [CO_2_], air temperature, and relative humidity in the greenhouse cells during the experiment period are shown in Fig. 7. All pots were well watered to 95% pot water holding capacity after seedling establishment.

### Measurements

#### Leaf gas exchange

Six weeks after sowing, net photosynthetic rate (*A*_n_), stomatal conductance (*g*_s_), and transpiration rate (*T*_r_) were measured on upper canopy fully expanded leaves (one leaflet per plant, eight WT and eight *flacca* plants per cell, respectively) between 9:00 to 12:00 using a portable photosynthetic system (LiCor-6400XT, LI-Cor, NE, USA). Measurements were done at 20 °C chamber temperature and 1200 mol m^−2^ s^−1^ PAR, and 400 ppm in cuvette for *a*[CO_2_] and 800 ppm in cuvette for *e*[CO_2_] growth environment, respectively.

#### Stomatal density

SSD was measured using a digital microscope (Dino lite AM4113/AD4113 series with ver. 1.4.1, Vidy Precision Equipment Co. Ltd, Wuxi, China). For each plant, three images (calibrated image size: 654 × 490 μm) from both the adaxial and abaxial leaf surfaces were taken (one leaflet per plant, eight WT and eight *flacca* plants per cell, respectively). ImageJ software (Version 1.51k, Wayne Rasband, National Institutes of Health, USA, Java 1.6.0–24 (64 bit)) was used for counting the stomatal number.

#### Plant water relations

Midday leaf water potential (*Ψ*_l_) was measured on young fully expended leaf (one leaflet per plant, eight WT and eight *flacca* plants per cell, respectively) using a scholander-type pressure chamber (Soil Moisture Equipment Corp., Santa Barbara, CA, USA). After measuring *Ψ*_l_, the leaf was immediately cut into two pieces and packed in aluminum foil separately and frozen in liquid nitrogen for later determination of leaf osmotic potential (*Ψ*_π_) and leaf ABA concentration ([ABA]_leaf_). *Ψ*_π_ was measured using a psychrometer (C-52 sample chamber, Wescor Crop, Logan, UT, USA) connected to a microvoltmeter (HR-33T, Wescor, Logan, UT, USA) at 22 ± 1 °C. Turgor pressure (*Ψ*_p_) was calculated as *Ψ*_l_ − *Ψ*_π_.

Leaf hydraulic conductance (*K*_l_, mmol m^−2^ s^−1^ MPa^−1^) was calculated as:

1 $$K_{\mathrm{l}} = \frac{{T_{\mathrm{r}}}}{{{\it{\Psi _{\mathrm{l}}}}}}$$

where *T*_r_ is the transpiration rate and *Ψ*_l_ is the leaf water potential.

Root water potential was measured on four WT and four *flacca* plants, respectively, in each greenhouse cell with a scholander-type pressure chamber (AGRSCI, KVL, Denmark). The whole pots were put into the chamber, then the chamber was sealed and only the above-soil part of the plants was left out. The stem was cut with a scalpel at approximate 10 cm above the soil surface. By pressuring the whole root system, the *Ψ*_r_ was determined when the xylem sap started to appear from the cutting surface. And the pressure was increased until it equaled *Ψ*_l_ of the plant to ensure a sap flow rate similar to the transpiration rate of the plant. Approximately 0.5–1 ml of xylem sap was collected to Eppendorf tubes using a pipette. Immediately after collection, the xylem sap was weighed and then frozen in liquid nitrogen and stored at −80 °C for ABA analysis. The time for collecting the sap was recorded and the stem cross-section area was measured. Then the hydraulic conductance of the whole root system (*K*_r_, g cm^−2^ min^−1^ MPa^−1^) was calculated as:

2 $$K_{\mathrm{r}} = \frac{{{\mathrm{Xylem}\,{\mathrm{mass}}}}}{{T\times P\times S}}$$

where xylem mass is the weight of the collected xylem sap (g); *T* is the collection time (s); *P* is the chamber pressure (MPa), which was maintained during collection; and *S* is the stem cross-section area (cm^2^).

Plant leaf area was determined by a leaf area meter (LICOR 3100, LI-COR Inc., Lincoln, NB) and the shoot biomass was determined after oven-drying at 70 °C for 48 h.

#### Xylem sap pH

After thawing for 30 min, the pH of the xylem sap was determined with a microelectrode (model PHR-146, Lazar Research Laboratories, Inc., CA, USA) interfaced with a pH meter (Model 60, Jenco Instruments Inc., CA, USA).

#### Leaf and xylem sap ABA concentration

Enzyme-linked immunosorbent assay was used to determine ABA concentration in the leaf and xylem sap samples following the protocol of Asch^53^. For the leaf ABA assay, we used the same leaf samples for determining *Ψ*_l_, which could have caused dehydration of the leaf thus affecting leaf ABA concentration. To clarify this, an extra test was done where ABA concentration of leaf samples from the same plants with and without *Ψ*_l_ measurements was compared, and no differences in ABA concentration were found between the two groups of leaves. Therefore, our method is valid for evaluating the leaf ABA concentration under the different treatments.

#### DNA/RNA extractions, cDNA synthesis, and PCR reactions

DNA and RNA extractions were done from 80 to 100 mg grinded leaf or root material using the DNeasy Plant Mini Kit or the RNeasy Plant Mini Kit, respectively, as recommended by the supplier (Qiagen, Germany). DNA or RNA yield and purity were estimated using Nanodrop^TM^ 1000 spectrophotometer (Thermo Fisher Scientific Inc., USA). RNA integrity was verified on agarose gels. Purified RNA was stored at −80 °C. For expression analyses, 1 µg of RNA was treated with DNase I Amplification Grade (Sigma-Aldrich, USA) and cDNA were synthesized using the iScript cDNA Synthesis Kit (Bio-Rad, USA) as recommended. cDNA was diluted fivefold in RNase/DNase free Tris-EDTA pH 7.4 (Sigma-Aldrich) for initial tests of PIPs in reverse transcriptase PCR). To target plasma membrane-localized aquaporins likely to transport water, the PIP subfamily were selected. Subsequently, tomato-specific PIP primers developed previously^54^ were used to pinpoint which PIPs where expressed in the tissues of this study. All initial PCR reactions using gDNA or cDNA were done using Ex taq polymerase (Takara Bio Inc, Japan) as recommended with 2% (v/v) dimethyl sulfoxide in final reactions. PCR conditions were 94 °C 4 min, 35 cycles of [30 s 94 °C, 1 min 60 °C, 45 s 72 °C], and 7 min 72 °C. Among the 12 PIPs tested (*PIP1.1*–*PIP1.3*, *PIP1.5*, *PIP1.7*, *PIP2.1*, *PIP2.4*–*PIP2.6*, *PIP2.8*, *PIP2.9*, and *PIP2.12*), 4 were not suitable for the subsequent quantitative PCR (qPCR) analyses. *PIP1.2*, *PIP2.6*, and *PIP2.12* were detected in very low abundances or were not expressed. *PIP1.7* was found to be highly unstable and were excluded from the analyses.

#### Quantitative real-time PCR analyses (RT-qPCR)

Reactions of RT-qPCR were performed using SsoAdvanced^TM^ Universal SYBR^®^ Green Supermix as recommended (Bio-Rad) with a CFX Connect^TM^ Real-Time PCR Detection System (Bio-Rad). Analyses of primer temperature optimization, melting curves, standard curves for primer pair efficiencies, Cq values, and normalized expression (Cq) were conducted in CFX Maestro Software supplied by Bio-Rad. In addition to PIP primer pairs, tomato-specific *TIP4.1*, *SAND*, *CAC*, and *Expressed* reference gene candidates developed elsewhere were included in the analyses^55^. CAC was selected as reference gene in RefFinder^56^. Primer-specific temperature settings and efficiencies are available in Supporting Information Table S1. Each treatment type were analyzed with three technical and four biological replicates. Changes to fold change less than twofold up or down were considered minor. The full RT-qPCR assay were conducted twice from the level of RNA extractions.

### Statistics

Data were statistically analyzed using Microsoft Excel, SPSS 22.0 software (IBM SPSS Software, New York, USA), and CFX Maestro Software (Bio-Rad). The effects of CO_2_ growth environment and GE and their interaction on variables were analyzed using two-way analysis of variance (ANOVA). In addition, in order to discriminate the means between the four treatments, one-way ANOVA (Tukey’s test) was conducted to determine the significant differences. Differences between treatments were considered significant when *P* < 0.05.

## Supplementary information

Supplementary Information
