# Supplementary material for: ABA-mediated regulation of leaf and root hydraulic conductance in tomato grown at elevated CO2 is associated with altered gene expression of aquaporins
Source: Hortic Res. 2019 Sep 11;6:104. doi: 10.1038/s41438-019-0187-6 (PMC6804533; doi:10.1038/s41438-019-0187-6)
Supplement: Supplementary file 1 — Supplementary Information [file 41438_2019_187_MOESM1_ESM.docx]

**Supplementary Information**

**Table S1.** Aquaporin and reference gene specific settings in quantitative real time PCR (RT-qPCR) runs and analyses. References for primer sequences are presented below.

| Target  gene | Temperature  in RT-qPCR  (°C) | | Sequence | Primer pair  performance | | |
| --- | --- | --- | --- | --- | --- | --- |
|  |  |  | | Efficiency | | R^2^ |
| *PIP1.1*^a^ | 60 | FWD 5’-GAAATCTTAGTGAGTGAGTGAG-3’ | | 101.1 | 0.998 | |
|  |  | REV 5’-ATGATGATAGTTCACCAGG-3’ | |  |  |  |
| *PIP1.3*^a^ | 60 | FWD 5’-GGCTACCATTCCAATCACCG-3’ | | 94.7 | 0.998 | |
|  |  | REV 5’-CAACAGCACCAGACAGG-3’ | |  |  |  |
| *PIP1.5*^a^ | 58.4 | FWD 5’- GCTGCTCTTGCTGCTATTT-3’ | | 99.6 | 0.998 | |
|  |  | REV 5’- CCTTCATTGATAAGGTACA-3’ | |  |  |  |
| *PIP2.1*^a^ | 60 | FWD 5’-GTGCTGCTGTTGTTTATGGACA-3’ | | 92.1 | 0.997 | |
|  |  | REV 5’-CATCCAACACAACTCTAACAAC-3’ | |  |  |  |
| *PIP2.4*^a^ | 60 | FWD 5’-CAATGGTGACAAGGCGTGG-3’ | | 100.3 | 0.998 | |
|  |  | REV 5’-GAAGGCGAATTCATAGGAT-3’ | |  |  |  |
| *PIP2.5*^a^ | 58.4 | FWD 5’-GGATATGGAGTATGGAAATG-3’ | | 97.5 | 0.999 | |
|  |  | REV 5’-TTGGTCACCATCACTTTG-3’ | |  |  |  |
| *PIP2.8*^a^ | 61.9 | FWD 5’-GGAGCTGCTGTTATTGCTGA-3’ | | 95.6 | 0.997 | |
|  |  | REV 5’-GCACAGATCCAAGGCTAAGA-3’ | |  |  |  |
| *PIP2.9*^a^ | 61.9 | FWD 5’-GCAATGGCAGCAGCAATATACCA-3’ | | 95.3 | 0.997 | |
|  |  | REV 5’-CGAAAGAGAATAGACCACCA-3’ | |  |  |  |
| *CAC*^b^ | 61.9 | FWD 5’-CCTCCGTTGTGATGTAACTGG-3’ | | 107.3 | 0.997 | |
|  |  | REV 5’-ATTGGTGGAAAGTAACATCATCG-3’ | |  |  |  |
| *SAND*^b^ | 58.4 | FWD 5’-TTGCTTGGAGGAACAGACG-3’ | | 101.1 | 0.997 | |
|  |  | REV 5’-GCAAACAGAACCCCTGAATC-3’ | |  |  |  |
| *TIP4.1*^b^ | 60 | FWD 5’-ATGGAGTTTTTGAGTCTTCTGC-3’ | | 100.7 | 0.998 | |
|  |  | REV 5’-GCTGCGTTTCTGGCTTAGG-3’ | |  |  |  |
| *Express-ed*^b^ | 60 | FWD 5’-GCTAAGAACGCTGGACCTAATG-3’ | | 99.1 | 0.996 | |
|  |  | REV 5’-TGGGTGTGCCTTTCTGAATG-3’ | |  |  |  |

^a^Reuscher, S., Akiyama, M., Mori, C., Aoki, K., Shibato, D., & Shiratake, K. (2013) Genome-wide identification and expression analysis of aquaporins in tomato. Plos One, 8, e79052.

^b^Expósito-Rodríguez, M., Borges, A.A., Borges-Pérez, A., & Pérez, J.A. (2008). Selection of internal control genes for quantitative real-time RT-PCR studies during tomato development process. BMC Plant Biology, 22, 131.


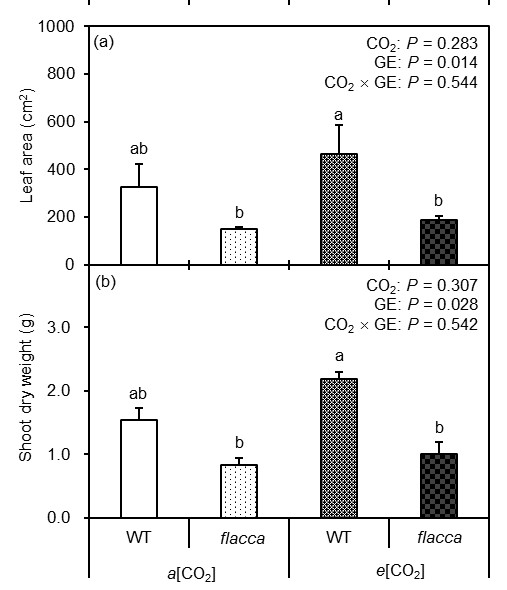


**Fig. S1.** Leaf area (a) and shoot dry weight (b) of wild type tomato ‘Ailsa Craig’ (WT) and its representative ABA-deficient mutant (*flacca*) grown under ambient (400 ppm, *a*[CO_2_]) and elevated (800 ppm, *e*[CO_2_]) atmospheric CO_2_ concentrations. Statistical comparisons (two-way ANOVA) between the CO_2_ growth environment (CO_2_) and genotype (GE) as well as their interactions CO_2_ × GE are presented. The different letters on the columns indicate significant difference at *P* < 0.05 level (one-way ANOVA).Error bars indicate standard error of the means (SE) (n=4).
